# Craniometric Measurements and Surgical Outcomes in Trigonocephaly Patients Who Underwent Surgical Treatment

**DOI:** 10.7759/cureus.13676

**Published:** 2021-03-03

**Authors:** Nuri Serdar Baş, Serap Baş

**Affiliations:** 1 Department of Neurosurgery, Bagcilar Education and Research Hospital, University of Health Sciences, Istanbul, TUR; 2 Department of Radiology, Gaziosmanpaşa Hospital, İstanbul Yeni Yüzyıl University, Istanbul, TUR

**Keywords:** trigonocephaly, craniometric measurement, metopic craniosynostosis, open reconstructive surgery

## Abstract

Objective

The aim of this study was to discuss the results of craniometric measurements and surgical treatments in patients operated for isolated trigonocephaly (TC) in light of the current literature.

Methods

A total of 18 cases who underwent surgery for isolated TC were included in the study. Age, gender, family history, follow-up time, complications, duration of surgery, surgical blood loss, and amount of blood replacement in these patients were recorded. Craniometric measurements such as metopic angle (MA), cephalic index (CI), interparietal distance (IPD), intercoronal distance (ICD), and their ratio to each other were evaluated according to pre-and postoperative parameters. Photographs of the patients were taken before and after the operation. The Whitaker classification and Kampf "aesthetic outcome staging" were used in the evaluations.

Results

The mean MA values after the operation increased to reach above 147 degrees in all cases. The average CI did not change. ICD measurement averages increased significantly. The average IPD/ICD ratio decreased due to the increase in ICD and the enlargement of the anterior fossa after the operation. According to the Whitaker classification and Kampf “aesthetic outcome staging” scale, 17 of our cases were at stage I, rated as perfect, and one was at stage II, rated as good.

Conclusion

Surgery performed at the appropriate time for TC yields cosmetically satisfactory results. Since potential neurological and cognitive morbidities occur mostly in school-age patients, long-term follow-up of the cases is required. Performing craniometric measurements enables patients to be evaluated with objective and measurable numerical data.

## Introduction

Trigonocephaly (TC) is a type of craniosynostosis (CS) that develops due to the premature fusing of the metopic suture. In this condition. the head has a triangular shape. The anterior corner of the triangle is formed by the metopic suture, and the compensatorily expanded parieto-occipital bones form the posterior two corners. Although the prevalence of TC was previously reported to range from 1/10,000 to 1/15000 births on average, its incidence has increased in recent years [1-4]. Approximately 20.1%-25.5% of all patients with synostosis have been reported to have TC [3,4]. Although its etiology is not exactly known, many different factors have been identified as potentially contributing to its occurrence. It is remarkable that there are clinical cases of TC that manifest not only the organic brain and mental problems but also psychological and behavioral problems. The treatment of the TC is surgical, and various surgical techniques have been described in the literature [3].

The aim of this study was to discuss the results of craniometric measurements and surgical treatments in patients operated for isolated TC in light of the current literature.

## Materials and methods

In our study, data related to the cases of CS, who were operated on by the same surgeon (NSB) between 2010 and 2020, were compiled and the patients who underwent surgery for isolated TC were assessed based on patient files and cranial CT examinations. Patients with non-isolated TC and non-TC CS were excluded from this study. Ultimately, 18 patients who underwent surgery for isolated TC were included. These patients were assessed based on the pre-and postoperative parameters of craniometric measurements such as age, gender, family history, the period of follow-up, complications, duration of surgery, surgical blood loss, blood replacement and amount, metopic angle (MA), cephalic index (CI), interparietal distance (IPD), intercoronal distance (ICD) as well as their ratios to each other, and the data were recorded (Table 1).

**Table 1:** Demography, type of surgery, operative data, pre-and postoperative craniometric measurements, hospitalization, and clinical follow-up time MA: metopic angle; CI: cephalic index; ICD: intercoronal distance; IPD: interparietal distance; intraop: intraoperative; preop: preoperative; postop: postoperative; ORS: open reconstructive surgery; EAS: endoscopy-assisted suturectomy

Specifications	Values
Demography	
Age (months) (n=18)	6.05 (1-9)
Gender	
M	14 (77%)
F	4 (23%)
Type of surgery (n=18)	
ORS	17
EAS	1
Operative data (average); operation time (minutes)	
ORS	166.1 (140-210)
EAS	55
Operative blood loss (ml)	
ORS	157.6 (120-200)
EAS	25
Transfusion (ml)	
ORS	107.6 (50-160)
EAS	-
Complication	
Intraop	-
Postop	
Local wound problem	1
Subgaleal hematoma	1
Intra-extracranial additional pathology	
Arachnoid cyst	2
Cavum septum pellucidum	1
Strabismus	5
Undescended testicles	2
Hypospadias	1
Craniometric measurements	
MA preop (degree)	118.7^0 ^(111^0^-128^0^)
MA postop (degree)	154.2^0^ (148^0^-163^0^)
CI preop	83.7 (76-92)
CI postop	85.5 (80-94)
ICD preop (mm)	86.6 (80-95)
ICD postop (mm)	107 (88-135)
IPD/ICD preop	1.36 (1.23-1.56)
IPD/ICD postop	1.11 (1.06-1.22)
IPD (mm)	118.6 (99-130)
Postop stay (days) (n=18)	7.1 (3-10)
ORS (n=17)	7.4 (5-10)
EAS (n=1)	3
Follow-up time (months)	18 (12-37)

Statistical analyses were performed using SPSS Statistics for Windows version 22.0 (IBM, Armonk, NY) and paired Student's t-test; a p-value of <0.05 was considered statistically significant. This study was performed in line with the principles of the Declaration of Helsinki. Approval was granted by the Ethics Committee of Istanbul Yeni Yüzyil University (Date: 07.12.2020; no. 2020/12-549).

Severity assessment of trigonocephaly

The severity of TC was assessed using MA measurements. Preoperative MA values were categorized as follows: severe (111^0^-123^0^), moderate, (124^0^-135^0^), and mild (136^0^-147^0^). An MA value of <147^0^ was considered normal [5]. The distribution of the patients by MA values is presented in Table 2.

**Table 2:** Preoperative MA values; distribution of patients according to postoperative Whitaker classification MA: metopic angle

Severity	MA staging	N (%)	Postoperative Whitaker classification			
			Grade I	Grade II	Grade III	Grade IV
Mild	136^0^-147^0^	-	-	-	-	-
Moderate	124^0^-135^0^	4 (22%)	4 (22%)	-	-	-
Severe	111^0^-123^0^	14 (78%)	13 (72%)	1 (6%)	-	-
Total		18 (100%)	17 (94%)	1 (6%)	-	-

Cranial CT craniometric measurements

Cranial CT (2D + 3D) was performed in all patients (SOMATOM Sensation 64, Siemens AG, Forchheim, Germany). Craniometric measurements on cranial CTs were performed on a CT workstation by an experienced radiologist (SB).

**Figure 1:**
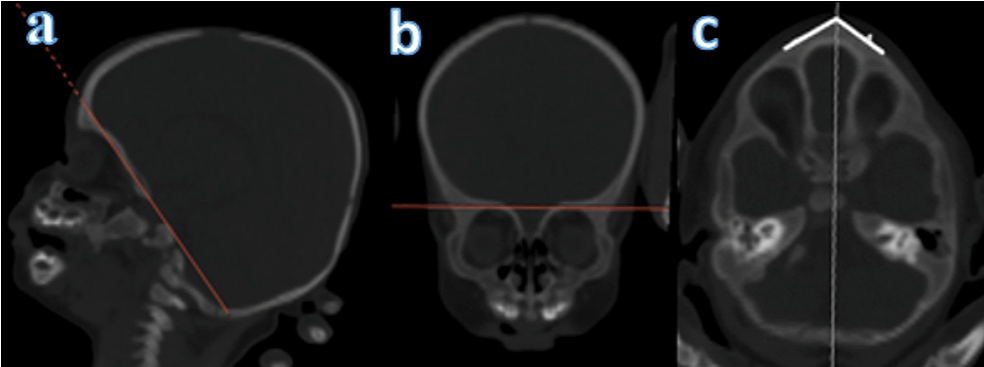
Metopic angle measurements in the supraorbital plane a: sagittal; b: coronal; c: axial

MA angle measurements in the supraorbital plane are presented below (Figure 1). MA is defined as follows: in supraorbital 2D axial CT sections, the angle in the axial section parallel to the orbital ceiling, the corner of which is at the most protruding point of the metopic ridge and the arms extending laterally on the frontal bone surface [5,6].

**Figure 2:**
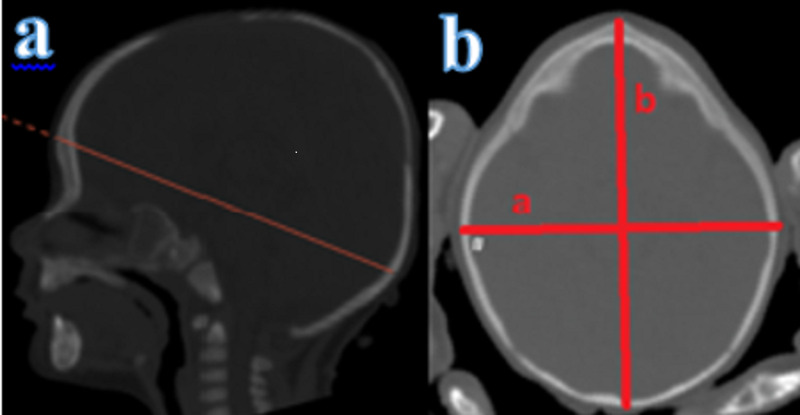
Cephalic index measurement a: cephalic index measurement plane in sagittal section; b: cephalic index measurement (a/b x 100)

CI measurement is illustrated below (Figure 2). CI is defined as the maximum cranial width/maximum cranial AP distance x 100.

**Figure 3:**
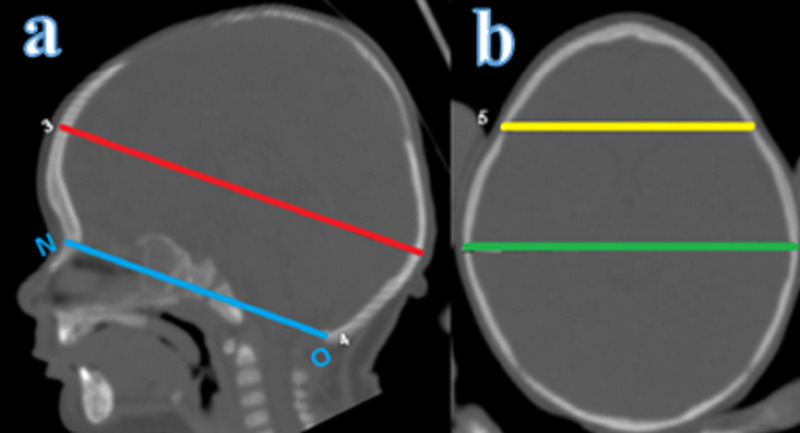
IPD and ICD measurement a: ICD and IPD measurement plane at foramen Monro level parallel to N-O plane (top line, red); b: ICD (anterior line, yellow) and IPD (posterior line, green) IPD: interparietal distance; ICD: intercoronal distance; N: nasion; O: opistion line

IPD and ICD measurements are depicted in Figure 3. IPD is defined as biparietal posterior maximum distance. ICD is defined as the distance between the outer surfaces of the coronal sutures.

IPD and ICD measurements were calculated using the plane obtained by shifting the plane passing through the nasion-opisthion line to the foramen Monro level in parallel (Figure 3) [5,6]. Measurements were taken separately for pre-and postoperative periods and compared using statistical analysis. Preoperative and postoperative results for MA, CI, ICD, IPD/ICD measurements are presented in Table 1.

Surgery and follow-up criteria

Endoscopy-assisted suturectomy (EAS) or open reconstructive surgery (ORS) was performed on the patients. The follow-up examinations of the patients were conducted in the first, third, sixth, and 12th months after surgery, and the subsequent follow-ups were performed in one-year intervals in the patients who were followed up. Short- and long-term follow-ups were performed by clinical examination and taking photographs, and the satisfaction levels of the families regarding the procedure were ascertained. Cranial CT was performed in the early postoperative period and during the first-year follow-ups of the patients. In the assessments, the Whitaker classification (Table 2) [7] and "aesthetic outcome staging" (Table 3) of Kampf et al. [8] were used. The Whitaker classification stages were categorized as follows - stage I: patients for whom surgical revision was not recommended or required; stage II: patients for whom minimal revision was recommended (in soft tissue or bone contours); stage III: patients for whom major revision was recommended (alternative osteotomies or bone grafting); stage IV: patients for whom major revision was recommended (craniofacial procedures exceeding the original surgery).

**Table 3:** Aesthetic outcome staging

Staging	Whitaker score	Symmetry	Surgeon's and parents' opinions	N
Stage I (excellent)	I	Yes	Adequate	17
Stage II (good)	I	No	Adequate	1
Stage III	I	No	Non-adequate	-
Stage IV	II + III + IV	No	Non-adequate	

## Results

All patients were aged between one and nine months (mean age: six months). There was a distinct male predominance [14 males (77%)] in the gender distribution. The M/F ratio was 3.5. Two of the patients were siblings.

Surgical treatments

**Figure 4:**
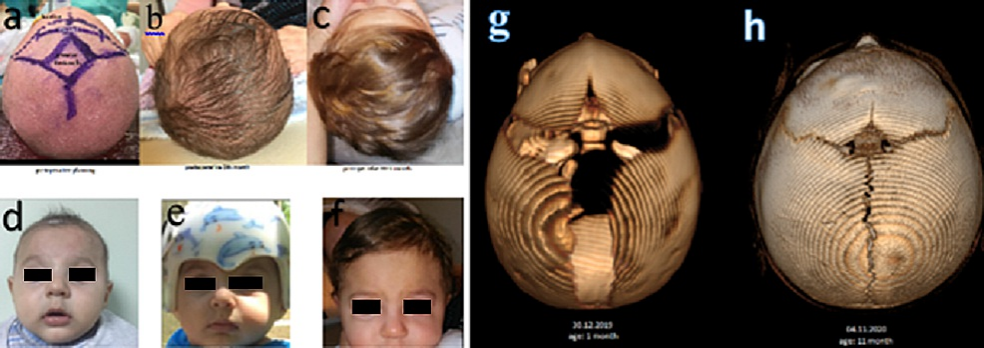
Endoscopy-assisted suturectomy Preoperative preparation (a), postop second-month (d), postop sixth-month (b,e) (helmet) and 11th-month (c,f) clinical appearance. Preop (g) and postop 11th-month (h) 3D CT CT: computed tomography

**Figure 5:**
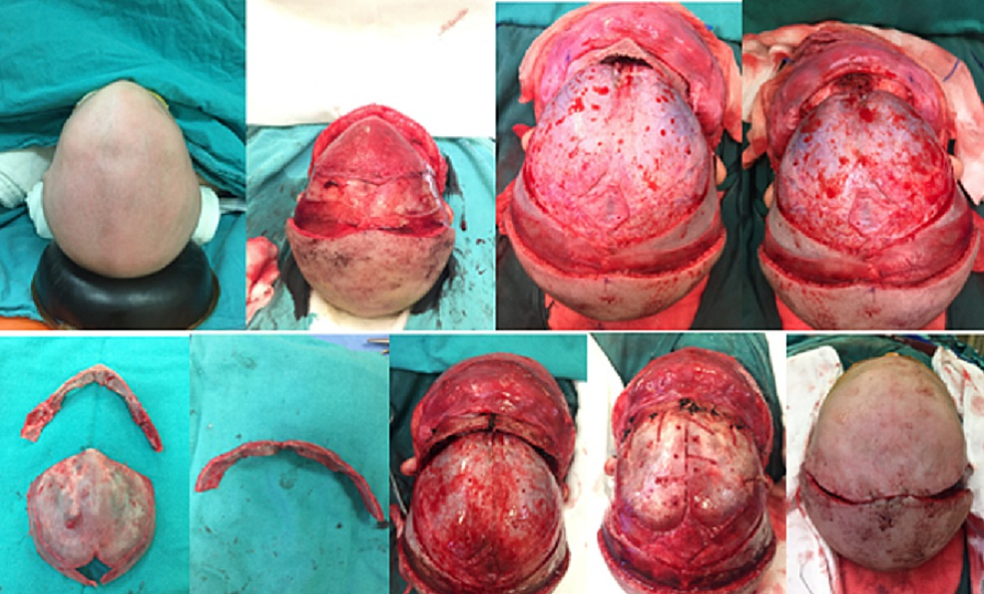
Open reconstructive surgery stages in trigonocephaly, per operative views

A patient diagnosed in the neonatal period was treated with EAS and subsequently with eight months of helmet application (Figure 4). ORS therapy was performed in all remaining 17 patients. In all our patients, 1% adrenaline xylocaine was applied to the skin at the incision line so that less bleeding was ensured. Following the bicoronal incision, the frontal flap was overturned anteriorly. Supraorbital nerves were preserved by a subperiosteal detachment of the periosteum. The fronto-orbital bar was removed, and the angular normalization of the midline was achieved, and the fronto-orbital bar was advanced with the "tongue in the groove" technique, and it was fixed again with suture or miniplate. Frontal bones were reshaped and fixed to the fronto-orbital bar using sutures and employing the "floating forehead" technique (Figure 5).

Cranial CT craniometric measurement results

In the preoperative assessment of MA measurement, 14 patients had severe TC, and four patients had moderate (almost severe) TC. There were no patients with mild TC in our series. The mean MA was 118.70^0^. The mean postoperative MA value increased to 154.20^0^. The mean preoperative and postoperative CI values were 83.70 and 85.50, respectively. The mean postoperative ICD measurement increased to 107 mm from the mean preoperative measurement value of 86.6 mm. While the mean preoperative IPD/ICD ratio was 1.37, it decreased to 1.12 due to the increase in ICD and the enlargement of the anterior fossa in the postoperative period.

Intra-extra cranial additional pathologies

Arachnoid cysts (AC) were detected with TC in two patients. An increase was detected in Galassi type I AC, which was in the left temporal region in one patient and bilateral temporally in the other, following the reconstructive surgery. One patient had cavum septum pellucidum (CSP). Regarding the presence of extracranial pathology, it was observed that five patients had minimal strabismus, one patient had hypospadias, and two patients had unilateral undescended testicles.

Complications

Superficial and localized wound infection (5%) was detected in one patient, who had been treated with antibiotic use, and subgaleal hematoma (5%) was detected in one patient. There were no severe complications or deaths in our series.

Final status and follow-up assessment

The patients had a follow-up period of 12 to 37 months (mean duration: 18 months). Patients were examined in the first, third, sixth, and 12th months and subsequently once a year. The Whitaker assessment results obtained during follow-ups are presented in Table 2. The other scale employed for the assessment was the aesthetic outcome staging, and the assessment criteria and results are presented in Table 3.

## Discussion

TC is caused by premature synostosis of the metopic suture, which is in the interfrontal zone, the midline between the nasion and bregma. Metopic suture is the earliest fused suture in infancy. The fusion of metopic suture is a process that normally begins in the third month after birth and lasts until the ninth month, which starts at the nasion and ends in the anterior fontanel. The severity of TC depends on the timing and extent of suture fusion. In TC patients, the tapering of the metopic suture towards the anterior, lateral inadequacy of supraorbital bar, hypoplasia of the ethmoid bone, and hypotelorism due to orbital medialization lead to a triangular shape in the head together with frontotemporal stenosis and widening of the biparietal diameter [2,3,9]. The anterior fontanel is fused prematurely in half of the cases [2,10].

The incidence of TC was previously estimated to range from 1/10,000 to 1/15,000 births on average. Recently, the incidence of TC has increased (1/5,000) due to an unknown cause and has become the second most common isolated single suture synostosis in series [2,3,11]. Di Rocco et al. have stated that there was a 420% increase in TC in the 20 years before 2009 [1]. It has been reported in the literature that the male dominance in TC is 3/1 [2,8,9,12,13]. In our series, the male-to-female ratio (M/F) was determined to be 3.5, which is in line with the literature. Although several hypotheses have been suggested regarding its etiology, the exact etiology is still unknown. In addition to hereditary factors, genetic mutations, metabolic, hormonal, and some genetic diseases, antiepileptic medications such as valproic acid [10], advanced maternal age, and smoking during pregnancy might play a role in the etiology [14]. TC patients with familial or autosomal dominant inheritance have been reported in the literature, as in the case of two brothers who were operated on in our study [13,15].

Surgery is the only treatment currently prescribed for TC. Technically, two primary methods are used: EAS and ORS. In open surgery, fronto-orbital advancement techniques, which increase the frontal fossa volume directly, are preferred. This technique provides opportunities such as 1) increasing the volume of the anterior cranial fossa, 2) reshaping of the frontal bone, 3) advancement of supraorbital bar 4), correction of hypotelorism, 5) temporal expansion of the skull, and 6) reorientation of skull growth vectors [16]. In our series, 94% of the patients underwent fronto-orbital advancement and calvarial remodeling procedures. The decision regarding which surgery to perform is predominantly related to the age of the patient. In the literature, EAS is preferred in patients aged four months and under [17,18].

All the patients who were operated on due to metopic synostosis were under 12 months, and the mean age was six months. In non-syndromic TC, the consensus is to operate within the first year after birth. In most of the published series, operations are recommended for patients aged under 12 months, particularly for those aged between three to nine months [2,3,8,19,20]. In our series, patients who would undergo reconstructive surgery were preferred to be operated on around the age of six months. We believe that the age of around six months is the optimal time for TC surgery due to worries about infants under six months having a lower tolerance to anesthesia and blood loss, an increase in the difficulty of bone reshaping because of the increased ossification in late-operated patients, and the likelihood of inadequate cosmetic satisfaction. Di Rocco et al. [16] have stated that for their technique, they preferred to wait until the patients reached the age of four months, enabling the maturity of the bone to gain stable structure; also, by the fourth month, fetal-type hemoglobin is reduced to the lowest level, and it is replaced by efficient adult-type hemoglobin, resulting in better surgical blood loss tolerance. Furthermore, it has been revealed in the literature that not removing the frontal pressure due to TC in the first year after birth, when rapid brain development occurs, may have adverse effects on IQ, mental and motor development, and orbital functions [2,3,8,19,20].

The biggest challenge in surgery for TC is the amount of bleeding. Patients undergoing EAS experience lower blood loss and hence require less transfusion [21]. Hinojosa revealed that blood loss and blood transfusion rates were lower among patients who underwent EAS [18]. Jimenez et al. compared 141 patients who underwent EAS with the values of the patients who underwent ORS in the literature and revealed that the mean blood loss of the patients who underwent this technique was 33 ml (224 ml in open surgery), and the blood transfusion rate was 4.3%, and these values were statistically significant [17]. Han et al. reported the blood loss in EAS to be 36.1 ml. In our series, only one patient underwent EAS, and the total blood loss in this patient was approximately 25 ml, and no transfusion was performed [22]. In previous studies, blood loss and transfusion rates in patients who underwent ORS are significantly higher compared to the endoscopic group and range from 224 ml to 400 ml [9,17]. In our series, the mean blood loss of patients who underwent ORS was 157 ml. All patients received perioperative and/or postoperative blood transfusion, with a mean of 107 ml. Our blood loss was lower compared to what is reported in the literature. The lower mean blood loss compared to the literature can be explained by the aggressive hemostasis that was started before the skin incision and was applied during the surgery and the use of an ultrasonic bone scalpel (Piezosurgery Plus, Mectron, Carasco, Italy) in cutting the fronto-orbital bar. In addition to shortening the duration of the operation, the use of an ultrasonic bone scalpel allows lower blood loss, better cosmetic improvement by making a thinner incision, and preservation of the dura and orbital structures during the supraorbital incision [23,24]. Moreover, in the assessment regarding the operation duration, the mean surgical duration was significantly shorter in patients who underwent EAS. The duration of the procedure ranges from 45 to 71 minutes for EAS [17,21,22,25], whereas the duration for the ORS procedure ranges from 141 to 224 minutes [17,22,25]. The duration in our study was 55 and 164.1 minutes, respectively, which is consistent with the literature.

In a study with approximately 300 patients conducted by Han et al., the duration of postoperative hospital stay of the patients was reported to be 1.1 days after EAS, while it was 3.8 days following ORS [22]. Jimenez et al. reported 1.7 and 3.7 days, respectively, in their study [17]. The postoperative hospital stay in our series was three days for EAS patients and 7.4 days for ORS patients. The total mean duration was calculated to be 7.1 days. The duration was slightly longer compared to the data revealed in the literature. The sociocultural levels of the families were low. Moreover, they were not able to care for a newly operated baby. Therefore, patients were hospitalized for a longer period of time.

In craniometric measurements, which were performed using cranial axial CTs, the mean MA increased from 118.7^0^ (SD ±5.26) to 154.2^0^ (SD ±4.27) postoperatively; hence, MA values of all patients increased to 1,470, which is considered normal. Hence, the tapering of the infants in the midline of the forehead was eliminated. The current change was statistically significant as well (p<0.0001). The preoperative and postoperative CI values were 83.7 (SD ±3.70) and 85.5 (SD ±3.51), respectively, and the operation did not lead to a significant change in this index. The difference was not statistically significant either (p=0.091). ICD results varied significantly; the mean value, which was 86.6 mm preoperatively, increased to 107 mm in the postoperative period (p<0.0001). This increase was also statistically significant. The preoperative and postoperative results for IPD/ICD measurements were 1.37 and 1.11, respectively (p<0.0001). An increase in ICD and decrease in IPD/ICD ratio verify the increase in anterior fossa size. Our results are also statistically significant and consistent with previous publications [5,6]. There was no statistically significant change in pre-and postoperative CI values since there was no significant change in the biparietal diameter and longest AP distance, which were assessed in the CI. This is in line with situations described in the literature [3].

AC as an additional intracranial pathology was detected with TC in two patients. One of the patients was detected with Galassi type I AC, which was in the left temporal region, while the other patient had Galassi type I AC located in the bilateral temporal region. Following reconstructive surgery, one patient had an increase in the size of the left temporal unilaterally located cyst, while the patient with a bilateral temporally located cyst had an increase in the size of the AC on the right side and a decrease in the cyst on the left. These patients are still being followed up. In a case presented in the literature regarding the coexistence of TC and AC, it was revealed that both two pathologies were intervened in the same session [26]. Wojcicki et al. revealed the presence of AC and CSP as concomitant intracranial pathology in their series but did not reveal any details about the cysts [2]. In our series, one patient had CSP. Regarding the presence of extracranial pathology, it was detected that five patients had mild strabismus, one patient had hypospadias, and two patients had unilateral undescended testicles.

In the assessment that was performed concerning surgical complications, superficial and localized wound infection (5%), which was treated with the use of antibiotics, were detected in one patient, while subgaleal hematoma (5%) was detected in another patient. The perioperative dural opening was primarily sutured in three patients (16%), and no cerebrospinal fluid (CSF) fistula occurred in the postoperative period. In the literature, complications related to cerebral contusion, stroke/intracranial hemorrhage, seizure, plaque-screw wounds, hypothermia, hyperthermia, cardiac events, sepsis, and blood transfusion have also been reported in general CS surgeries [27,28]. Moreover, Esparza et al. have reported two deaths in a series of 283 cases, including all isolated and syndromic operated craniosynostoses, and the mortality rate was 0.7% [28]. There was no death in our series.

In this study, 14 (77%) of the patients we operated on with the diagnosis of TC had a severe condition. The severity of the patients leads to a significant difference in the appearance of the infants between pre-and postoperative periods. It has been observed that the significant changes, which occurred in the head and face appearance between pre-and postoperative periods, met the cosmetic correction expectations of the families. The final assessment of the patients was made in accordance with the Whitaker classification [7]. The results in our patients regarding these assessments are shown in Table 2. In the Whitaker assessment of the patients, it was determined that only one patient had a condition that might require minor revision. However, no additional surgery was planned as the family did not have any complaints regarding the current situation and their satisfaction with the surgery was high.

Kelleher et al. [29] have revealed a higher incidence of developmental, educational, and behavioral problems in isolated TC patients compared to other types of synostosis. Their findings showed that 34% of the patients had speech and language retardation, and 33% needed an educational psychologist, while 20% of them needed classroom assistant, and 47% needed support within the school system. However, the results demonstrated that there was no difference between children who were treated surgically and those with mild deformities who were treated conservatively. The fact that neurodevelopmental delays are observed in both patients with milder TC that have not been operated on and patients with TC who have undergone surgery suggests that the problem could be arising from the pathologies in the brain and are not a direct impact of growth-restricting CS [29,30].

The neurocognitive function and development of our patients during the current follow-up period were compatible with their age, and their developmental neurology examinations were within normal limits. As the literature corroborates, since all patients were operated on in the early period, we think that providing a space for rapid brain development in time promotes a favorable prognosis in our patients. Nevertheless, the shortness of the follow-up period and not continuing with the follow-ups until the patients reach an age when delays related to basic cognitive and learning can be detected are among the handicaps of this study, because most of the neurodevelopmental delay problems do not occur until the children reach school-age when they are more positioned to have social interactions and have higher expectations regarding the same with the intellectually challenging environment [3,30].

Limitations

The limitations of this study were the small number of patients and the fact that the follow-up periods did not continue until the school-age when the main intellectual retardation in the patients is detected. Studies involving a larger number of patients and longer follow-up periods could lead to more significant findings regarding neurodevelopmental assessments of patients with TC and secondary deformities that may occur at a later stage.

## Conclusions

TC is a congenital head shape anomaly that needs to be treated surgically, and cosmetic correction and adding volume to the frontal brain structures are the ultimate goals of the treatment. Endoscopic or open reconstructive surgery that is performed at the appropriate time yields cosmetically satisfactory outcomes. Long-term follow-up of the patients is of utmost importance, as potential neurological and cognitive morbidities mostly occur when the children reach school-age. Performing craniometric measurements enables patients to be assessed based on objective and measurable numerical data. Moreover, positive changes in measurements can be used as mathematical equivalents and evidence of satisfactory outcomes.
